# Ligament Reconstruction for Recurrent Anterior Dislocation of the Radial Head

**DOI:** 10.1155/2019/6067312

**Published:** 2019-12-16

**Authors:** Taku Hatta, Kiyotsugu Shinagawa, Kou Hayashi, Kazushige Hasegawa, Yoshinori Miyasaka, Nobuyuki Yamamoto, Eiji Itoi

**Affiliations:** ^1^Department of Orthopaedic Surgery, Tohoku University School of Medicine, Japan; ^2^Department of Orthopaedic Surgery, Senen Rifu Hospital, Japan

## Abstract

Isolated recurrent dislocation of the radial head (RH) is very rare, and there have been few reports describing the surgical treatment of this injury. We herein report the case of a 13-year-old girl who underwent ligament reconstruction surgery for isolated recurrent RH dislocation. Her symptoms included pain and apprehension at the elbow with the forearm in supination. A radiologic examination revealed anterior dislocation of the RH with the forearm in supination but complete reduction with the forearm in neutral to pronated positions. Surgical treatment to reconstruct the annular ligament and facilitate the radial collateral ligament was performed using an autograft with internal brace augmentation. At a 12-month follow-up examination, the patient had asymptomatic stability with recovery to sports activities. This case report describes a novel technique for the treatment of a rare pathological condition of the elbow.

## 1. Introduction

Isolated traumatic dislocation of the radial head (RH) is a rare injury [1, 2]. It has been reported this injury may occur due to a unique dislocation mechanism in which the elbow joint is subjected to varus stress with a specific position in which the forearm is pronated and the elbow is slightly flexed [3]. The cadaveric study suggested the ligamentous rupture could be caused by this injury mechanism, and various directions of RH dislocation could be associated with the arm positions after the injury. A recent biomechanical study also investigated the potential for combined ligamentous rupture to cause RH dislocation [4].

On the other hand, isolated recurrent dislocation of the RH is very rare, and there have been few case reports that described the clinical outcomes after surgical treatment. We herein present a case report of a patient who had recurrent RH dislocation and who was treated with reconstruction of the lateral collateral ligament complex using the palmaris longus tendon.

## 2. Case Report

A 13-year-old right-handed girl presented to our outpatient clinic with motion pain and apprehension of her right elbow. The symptoms included intense pain with apprehension at the elbow when the forearm was supinated, which had been present since playing basketball 1 week earlier. On the other hand, asymptomatic stiff prominence at the anterolateral aspect of her right elbow had been recognized for 3 years. The painless phenomenon had been present when the forearm was supinated and absent when the forearm was in neutral to pronated positions.

**Figure 1:**
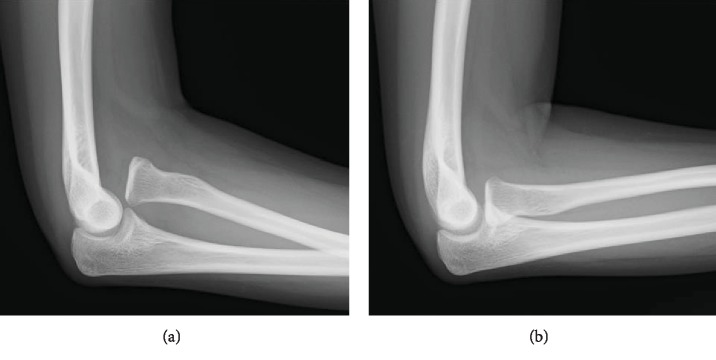
Preoperative radiographic images. The lateral radiographs show anterior dislocation of the radial head (RH) with the forearm in supination (a) and complete reduction of the RH in pronation (b).

**Figure 2:**
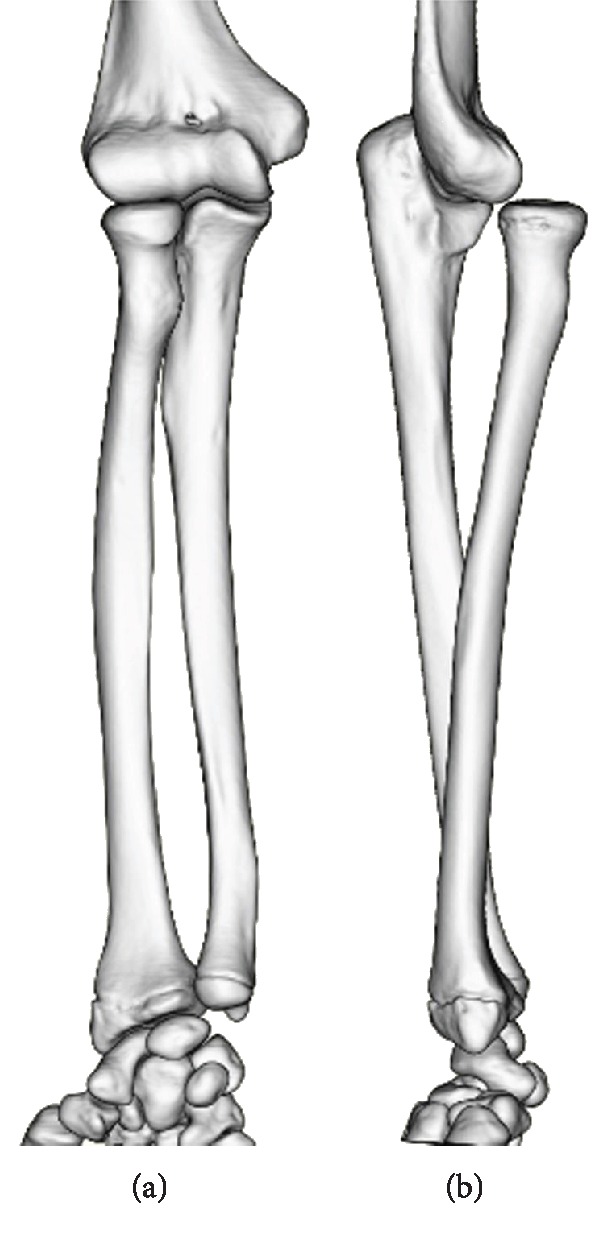
Three-dimensional computed tomography images with supinated forearm ((a) anteroposterior, (b) lateral). Isolated anterior dislocation of the RH is observed.

**Figure 3:**
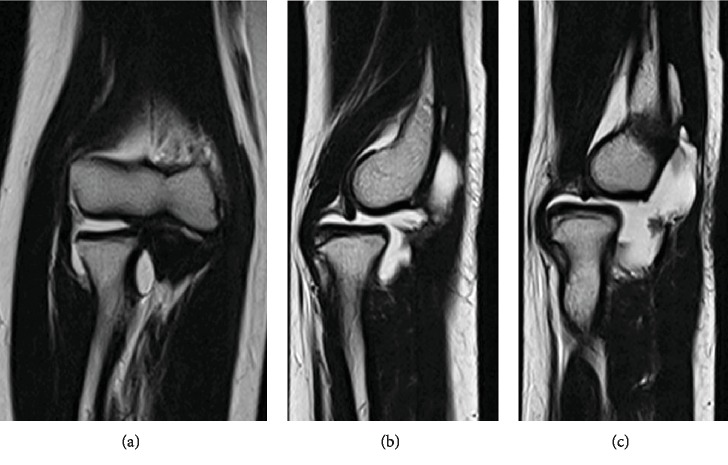
Preoperative magnetic resonance arthrography images ((a) coronal, (b, c) sagittal). The absence of the annular ligament is observed.

A physical examination revealed anterior dislocation of the RH when the forearm was supinated to an angle of approximately 30° or more, regardless of the elbow position (flex-extension). There were no deficits in the ranges of motion (ROMs) of the elbow and forearm. No general joint laxity was found. Plain radiographs (Figures 1(a) and 1(b)) and computed tomography (Figures 2(a) and 2(b)) performed at our clinic revealed isolated RH dislocation without deformity at the radius or ulna. Notably, the RH was anteriorly dislocated with the forearm in supination; whereas, complete reduction of the RH to the intact position could be obtained in neutral to pronated forearm positions. Magnetic resonance arthrography revealed the absence of the annular ligament (Figures 3(a)–3(c)).

Surgical treatment was performed under a diagnosis of recurrent RH dislocation due to insufficiency of the lateral collateral ligament complex. With tourniquet control, a 6 cm skin incision was created at the lateral elbow. Through Kocher's approach, the absence of the annular ligament in contrast to the continuity of the lateral ulnar collateral ligament (LUCL) was identified. The proximal region of the LUCL corresponding to part of the radial collateral ligament (RCL) was found to be relatively thin and fragile. The RH was easily dislocated with the forearm in supination; however, in neutral to pronated positions, the RH was reduced with suppression by tension of the supinator muscle.

**Figure 4:**
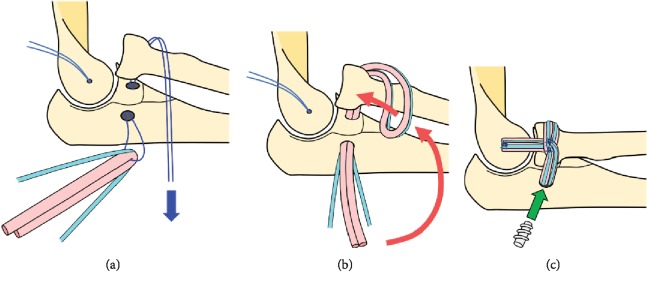
Schematic illustrations of ligament reconstruction surgery. After creating a tunnel at the proximal ulna, the loop of a number 2 high-strength suture is advanced through the tunnel (a). Autograft and a broad high-strength suture are shuttled through the loop to reconstruct the annular ligament (b). After fixation using an interference screw, the end of the graft complex is advanced proximally and fixed using a soft anchor placed at the lateral epicondyle (c).

We performed surgical treatment with a focus on the reconstruction of the annular ligament (Figures 4(a)–4(c)). We also aimed to facilitate the RCL because of potential weakness against varus stress. We harvested the plantaris tendon (width, 3 mm; length, 20 mm) from the ipsilateral leg. We decided to harvest the tendon because the palmaris longus tendon had been found to be of insufficient width and length for grafting according to preoperative sonographic examination. Moreover, we used 1.3 mm SutureTape (Arthrex, Naples, FL, USA) to increase the initial strength of the reconstructed regions, according to the concept of internal brace augmentation. A bony tunnel was created with a 3.5 mm cannulated drill at the radial aspect of the proximal ulna, and the loop of a number 2 FiberWire (Arthrex) was advanced through the tunnel. Then, the graft complex composed of the autograft tendon and the SutureTape were shuttled through the loop. After completing double-bundled reconstruction of the annular ligament, the graft complex was fixed to the tunnel using an interference screw (3 × 13.5 mm SwiveLock, Arthrex). The end of the graft complex was advanced proximally and fixed to the lateral condyle using a soft anchor (FiberTak, Arthrex) that had been placed at the region where the RCL attached.

**Figure 5:**
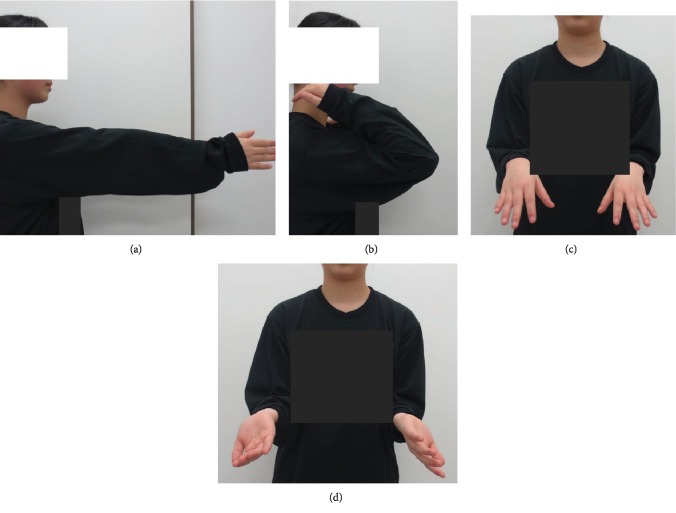
Ranges of motion at 1-year follow-up. Elbow extension (a) and flexion (b). Forearm supination (c) and pronation (d).

**Figure 6:**
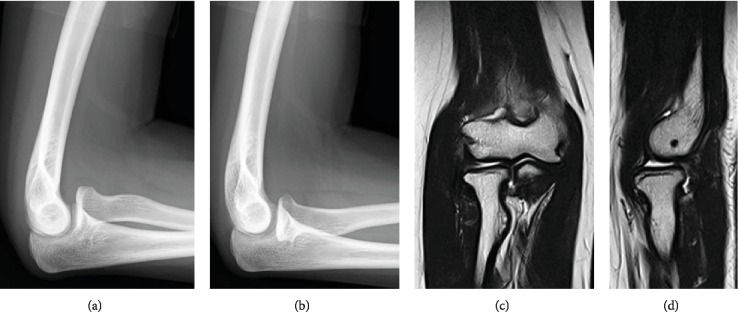
Radiographic images at 1-year follow-up. The lateral radiographs ((a) supination, (b) pronation) and T2-weighted magnetic resonance images ((c) coronal, (d) sagittal) show the reduced RH with reconstructed annular ligament.

Postoperatively, the patient was immobilized with a long-arm spica orthosis for 2 weeks and performed ROM exercises that were taught by a therapist. Daily activities were encouraged after the removal of the orthosis. However, the patient was instructed to avoid excessive exercise that supinated the forearm and to avoid returning to sports until 6 weeks and 3 months after surgery, respectively. At a 12-month follow-up examination, the patient had no pain or elbow limitation (Figures 5(a)–5(d)). The patient successfully returned to sports. Plain radiographs and magnetic resonance imaging (Figures 6(a)–6(d)) showed that the reconstructed annular ligament could support the position of the RH.

## 3. Discussion

We reported a very rare case of isolated recurrent RH dislocation. To our knowledge, there is only one other case report describing the same injury pattern [5]. Chronic posterolateral rotatory instability (PLRI) is known as the most common pathological condition causing recurrent subluxation or dislocation of the elbow joint. Disruption of the LUCL through mechanisms such as falling on the hand with the arm in an outstretched position can cause chronic PLRI after injury. Symptoms of chronic PLRI may include lateral-sided elbow pain and instability due to posterior translation of the RH and subsequently the ulna. In patients with isolated recurrent RH dislocation, in contrast, the RH is translated anteriorly, depending on the forearm position. Although the exact pathogenesis remains unclear, a recent biomechanical study investigated the injury mechanisms of ligamentous structures that cause anterior dislocation [4]. According to their cadaveric study, the complex of ligamentous structures comprising the annular and quadrate ligaments and the proximal part of the intraosseous membrane may play an important role in preventing anterior, posterior, and lateral dislocation of the RH. Notably, the most severe rupture patterns involving these three structures may be needed to cause dislocation in the anterior direction. This complicated injury mechanism for anterior RH dislocation may be the reason for the low prevalence of this injury pattern. In addition, a previous case report suggested that the contraction of the biceps brachii muscle causes anterior RH dislocation in specific cases in which dislocation occurs only with the forearm in supination [5]. We also hypothesized that increased passive stiffness of the supinator muscle due to the stretched muscle fibers might prevent anterior dislocation of the RH with the forearm in pronation. On the other hand, the current case did not undergo radiographic assessment for the contralateral side of the elbow or forearm. Although there were no obvious osseous deformities in involved side, radiologic assessment for the bilateral sides would be useful to determine the presence or absence of the deformity.

In the current case, we adopted a novel surgical technique for ligament reconstruction. Although there have been few reports describing surgical treatment for this rare injury pattern, the applicable options may include osteotomy of the ulna or radius, and/or reconstruction of the ligamentous structures. Itadera and Ueno [5] reported a surgical technique for reconstructing the annular ligament using the palmaris longus tendon. Similar to the current case, they treated a 16-year patient without obvious deformity of the bony structures. They indicated the advantage of their technique, including anatomical reconstruction of the ligamentous structures without nonanatomical transposition of the bones. In the present case, which involved a young patient who participated in sporting activities, we adopted their reconstructive surgery technique to achieve functional recovery without muscular imbalance. We modified the ligament reconstruction to facilitate the RCL as well as the annular ligament using a plantaris tendon, which might be longer than the palmaris tendon. Moreover, we applied the concept of internal brace augmentation for the reconstruction of the annular ligament and RCL to improve the initial strength of the autograft tendon using SutureTape. We believe that the use of this combined graft might facilitate effective reconstruction with the prevention of graft elongation, which has the potential to cause insufficient stability, according to the concept of internal brace augmentation. This concept was recently utilized for ligament repair and reconstruction using ligaments such as the scapholunate ligament, intermetacarpal ligament, anterior talofibular ligament, and the medical collateral ligament of the elbow [6–9].

## 4. Conclusion

We reported the case of a 13-year-old girl who underwent ligament reconstruction surgery for isolated recurrent RH dislocation. This case report describes a novel technique for the reconstruction of the annular ligament and facilitation of the RCL using an autograft with internal brace augmentation.
